# Bone mineral density and sex hormone binding globulin as potential mediators of the causal effect of urolithiasis on osteoporosis risk: a Mendelian randomization

**DOI:** 10.3389/fendo.2025.1460682

**Published:** 2025-02-26

**Authors:** Jiawei Guo, Xinyu Chen, Xinping Yi, Yongqi Dou, Yongjiang Xiong, Tao Zhao

**Affiliations:** Department of Urology, Yongchuan Hospital of Chongqing Medical University, Chongqing, China

**Keywords:** urolithiasis, osteoporosis, two-sample mendelian randomization, mediation analysis, casual association

## Abstract

**Objective:**

Osteoporosis (OP) and urolithiasis (UL) are two metabolic diseases that are prevalent globally. Previous observational studies have found a relationship between these two diseases that increases the risk of each other, but whether there is a direct causal link is still unclear. Currently, research on the mechanisms of these two diseases mainly focuses on external factors such as diet and environment. Thus, this study used two-sample mendelian randomization (TS-MR) in conjunction with mediation analysis to explore the causal relationship between OP and UL and their potential mechanisms. Mediators included total body bone mineral density (T-BMD), sex hormone binding globulin (SHBG), serum 25-hydroxyvitamin D (serum 25(OH)D) levels, and calcium supplements.

**Method:**

We acquired UL-related and BMD-related single nucleotide polymorphisms (SNPs) from the MRC IEU Open Genome-Wide Association Study (GWAS) database. The primary SNPs data of osteoporosis were from the FinnGen database. To clarify the mediators involved in the link between OP and UL, we performed a MR investigation. The primary approach to analysis was inverse variance weighting (IVW). In addition, we also used another osteoporosis data from UK biobank (UKB) to further verify the mediating role.

**Results:**

We discovered that there was a 14% increase in the incidence of OP in UL patients using the IVW approach. (FinnGen: OR = 1.1491,95% CI: 1.0544-1.2523; UKB: OR = 1.1339,95% CI: 1.0266-1.2523). Among different age groups, except for the 15-45 age group, we observed that UL increased the risk of low bone mineral density. Similarly, consistent results were also observed in bone mineral density at different sites. Mediation analysis showed that 50% of the effect of UL on OP was mediated by BMD levels (FinnGen:49.68%; UKB:56.45%). In addition, we also observed an important mediating effect between sex hormone binding globulin (SHBG) on UL and an increased risk of OP, but with a lower proportion of mediators (FinnGen:2.406%; UKB:2.595%). Furthermore, we also found decreased serum 25 (OH) D levels in UL patients, but not its mediating effect.

**Conclusions:**

In conclusion, the study establishes a direct causal link between urolithiasis and OP, independent of environmental factors. Furthermore, mediation analysis showed that bone density and SHBG levels partially mediated the risk of OP in UL patients, suggesting that both mediators may be involved in the mechanism of UL-induced OP. These findings broaden the understanding of the link between the UL and the OP. Thus, regardless of lifestyle, urolithiasis patients should remain vigilant about the risk of OP and consider regular OP screening.

## 1 Introduction

Osteoporosis (OP) is the most common metabolic bone disease today, characterized by low bone mass and deterioration of bone microstructure (1). Epidemiological estimates indicate that over 50% of women and 30% of males suffer with OP (2). Furthermore, due to increased population ageing, the prevalence of OP is expected to rise significantly, creating a heavy global health burden (3, 4). Overall, 60% of OP cases are caused by primary OP due to the aging process, while 40% are derived from other diseases. It is therefore important to figure out the risk factors of osteoporosis and discover the relationship between them.

Urolithiasis(UL) is one of the most common diseases in urology, affecting about 15% of the world’s population (5, 6). In recent years, the prevalence and incidence of UL have increased in many countries. Because UL and OP share common risk factors and are both metabolic diseases, there is increasing evidence that UL is associated with OP (7, 8). A large cohort study has shown that adults with OP have a higher risk of kidney stones (8). Similarly, this study also showed that adults with kidney stones have a higher risk of OP, regardless of age, gender, income, and area of residence (8). According to previous studies, the main etiology of the relationship between kidney stones and osteoporosis may be hypercalciuria and dietary calcium intake (9). It has been proposed that calcium and vitamin D supplementation may provide a modest reduction in fracture risk (10). The hypercalciuria was found to be linked to low serum vitamin D levels (11). The last metabolite of vitamin D is serum 25(OH)D (12). In addition to being engaged in hormone secretion and activation and preserving normal blood calcium levels, vitamin 25 (OH) D is essential for bone health (13). Clinically, vitamin D intake and use are frequently assessed using 25 (OH) D. In addition, several studies have shown that higher SHBG levels are associated with decreased BMD in both sexes (14, 15). Moreover, Reza Naghii et al. reported that serum SHBG was significantly increased in patients with kidney stones (16). Of course, bone mineral density and age remain important risk factors for OP. However, these studies were vulnerable to unmeasured confounding variables and reverse causation, which made it difficult to accurately determine the causative relationship between the two diseases (17). Mendelian randomization (MR) uses single nucleotide polymorphism (SNP) as a representative of exposure, which can minimize residual confusion in observational studies, thereby enhancing exposure and outcome causal inference (18). Therefore, through two-way Mendelian randomization and mediation analysis, we aim to elucidate the causal relationship and possible mechanism of action between UL and OP. Mediators included total body bone mineral density (T-BMD), sex hormone binding globulin (SHBG), serum 25-hydroxyvitamin D (serum 25(OH)D) levels, and calcium supplements.

## 2 Materials and methods

### 2.1 Study design

**Figure 1:**
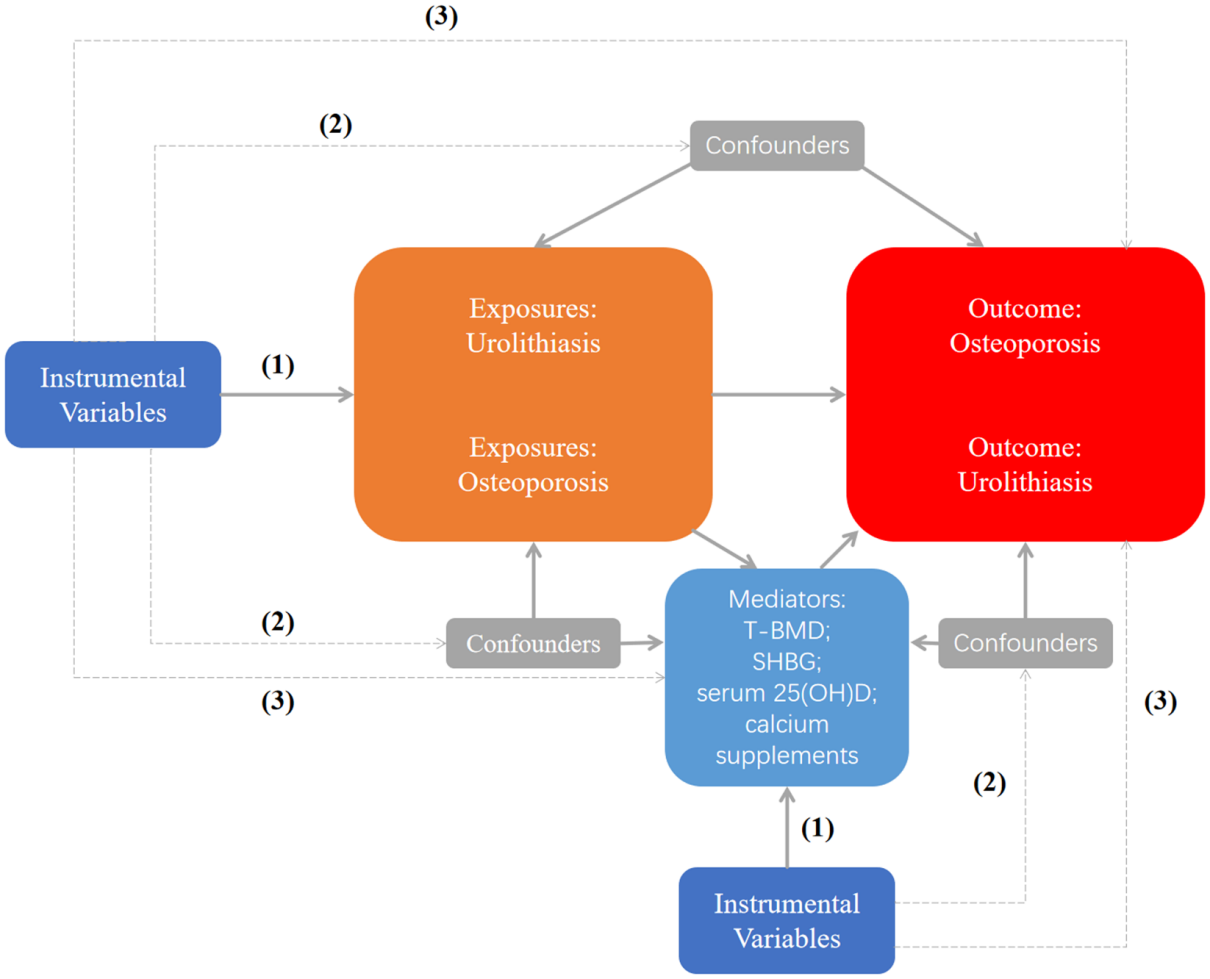
Study flow chart. (1) The IVs should have a significant correlation with exposure; (2) The IVs should be unaffected by confounding variables; (3) The IVs affect the outcome only through exposure. Dashed lines indicate no correlation, and solid lines indicate the correlation. T-BMD, total body bone mineral density; SHBG, sex hormone binding globulin.

This is a bidirectional two-sample and two-step MR study designed to investigate the causal relationship and potential mechanisms between UL and OP. The specific analysis steps are as follows: (1) to explore the causal relationship between UL and OP; (2) to assess the UL and BMD causal connection; (3) to study the potential mechanism of UL and OP. In the TS-MR analysis, single-nucleotide polymorphisms (SNPs) used as the instrumental variants (IVs) should satisfy three essential criteria: (1) They should have a significant correlation with exposure; (2) They should be unaffected by confounding variables; (3) They affect the outcome only through exposure (19). MR design can offer accurate causal effect estimates and control for any potential confounding factors only when all three requirements are satisfied, demonstrating the causal links between the two (20). No further ethical approval was required because all the data used had already been made available in the public database. The specific research diagram is shown in **Figure 1**. Age and bone mineral density have been demonstrated to be significant risk factors for OP. In childhood, bone mass increases gradually; in adolescence, it increases quickly, peaks at the age of 30, and then stays steady (21). After that, it started to steadily decline around the age of 50 and drastically dropped in postmenopausal women (22). The gold standard for determining bone mineral density levels is dual-energy X-ray absorptiometry, or DXA. In order to diagnose osteoporosis in postmenopausal women and men, the three most often utilized measurement sites are the femoral neck (FN), lumbar spine (LS), and forearm (FA). Therefore, in view of the relationship between UL and OP, we further explore whether UL still has a causal relationship with BMD levels in various age brackets and sites. We chose the largest dataset of total body bone mineral density(T-BMD) for various age groups as the outcome (23). The dataset comprises 66,628 individuals, categorized into five age groups: 0–15 years old, 15–30 years old, 30-45 years old, 45–60 years old, and older than 60. BMD in different parts includes bone mineral density of FA, FN and LS (24). Finally, in order to study the potential mechanism between UL and OP, we selected several possible mediators, namely T-BMD (23), serum 25(OH)D (25), calcium supplements and sex hormone binding globulin(SHBG) (26). Studies have shown that calcium and vitamin D supplementation can reduce the risk of OP (10). In addition, a number of studies have shown that higher SHBG levels are associated with OP (14, 15). Therefore, we selected these four possible mediation factors for mediation analysis (see **Supplementary Table S1** for specific data set information).

### 2.2 Data sources

All the related data for SNPs are from the open genome-wide association studies (GWAS). As aggregated data on exposure UL, download at GWAS ID:ebi-a-GCST90018935,which contained 6,223 cases and 482,123 control samples (27). For OP, we obtained pooled statistics from the FinnGen (8017 cases and 391037 controls) and the UK Biobank (UKB) database (6484 cases and 401279 controls) for the largest GWAS study (28, 29). For this research, we acquired genome-wide association study (GWAS) aggregate data on bone mineral density (BMD) from the GEnetic Factors for Osteoporosis Consortium website (GEFOS),including FA-BMD (GWAS ID: ieu-a-977), FN-BMD (GWAS ID: ieu-a-980), LS-BMD (GWAS ID: ieu-a-982),T-BMD (0–15)(GWAS ID: ebi-a-GCST005345), T-BMD (15–30)(GWAS ID: ebi-a-GCST005344), T-BMD (30–45)(GWAS ID: ebi-a-GCST005346), T-BMD (45–60)(GWAS ID: ebi-a-GCST005350), and T-BMD(>60)(GWAS ID: ebi-a-GCST005349). In addition, four mediators were derived from the European population: T-BMD(GWAS ID: ebi-a-GCST005348), SHBG(GWAS ID: ebi-a-GCST90012111), serum 25(OD)D(GWAS ID: ebi-a-GCST90000618), and Calcium supplements(GWAS ID: ukb-a-495). In this study, the disease cases cited conform to the International Consensus Standards (ICD-10). The case group is based on a comprehensive set of criteria, including diagnoses from medical registries, discharge summaries, and other relevant medical records. To ensure effective comparison, the control group consists of individuals from the general population who do not have the disease of interest. These control groups are precisely matched with the cases based on key demographic variables—age, gender, and ethnicity—to minimize potential confounding effects.

### 2.3 Selection of genetic instrumental variables

SNPs (p<5×10^−8^) that were substantially linked with UL were found in this investigation using GWAS aggregated data. To acquire a suitable number of SNPs in reverse MR, we change the p value of OP to 5×10^-6^. Then, in order to get independent instruments, relevant SNPs were clustered using linkage disequilibrium (LD) with R^2 < 0.001 and cluster distance (kb) = 10,000. The instrumental variables’ strength was assessed using F statistics (30). The weak instrumental variable is defined by the threshold F<10, so the deviation it causes can be disregarded. We harmonized the SNPs (31) after using PhenoScanner (32) to look for the relevant SNPs’ phenotypes, excluding any SNPs that might cause OP (Such as body mass index, alcohol, SHBG, serum 25(OH)D, and calcium, et al).Palindrome SNPs were also removed in this process. Ultimately, the remaining SNPs were chosen as IVs for the subsequent MR examination. The SNPs features included in this study are listed in **Supplementary Tables S2**–**S5**.

### 2.4 Statistical analysis

We employed four methods, primarily inversevariance weighted (IVW) methods, to determine if UL and OP are causally related (33). The remaining three methods include: MR Egger (34), weighted-median (35) and weighted mode (36). The following guidelines are adhered to: (1) When pleiotropy and heterogeneity are absent, IVW is the most dependable result to consider as a top priority; (2) When merely heterogeneity exists, the result of the preferred weighted medium method and IVW (multiplicative random effects); (3) The MR Egger method’s calculations yield better results when multiple validity is present (37). In addition, a number of sensitivity studies were performed to assess the association’s strength. Initially, funnel plots and the Cochran’s Q test were used to evaluate heterogeneity (38). Second, we used MR-Egger regression to determine whether the intercept was statistically different from zero to identify the presence of directed pleiotropy. Third, we confirmed the robustness of the results by applying the Leave-one-out approach (39). Fourth, we use the MR pleiotropy residual sum and outlier (MR-PRESSO) test to look for potential outliers (40). We presented the relationships between UL and the risk of OP using odds ratios (OR) along with their 95% confidence intervals (CI). According to the formula (Beta1 × Beta2)/Beta, the mediating ratio was calculated. With the aid of the related R packages “mrpresso1.0” and “TwoSampleMR0.5.8” and their dependent expansions, the aforementioned study is examined and displayed in R v.4.3.0. A statistically significant difference in this study was defined as p<0.05. This investigation adheres to the Strengthening the Reporting of Observational Studies in Epidemiology (STROBE) guideline.

## 3 Results

### 3.1 Bidirectional two-sample MR analyses

#### 3.1.1 Causal effects of UL on OP

**Figure 2:**
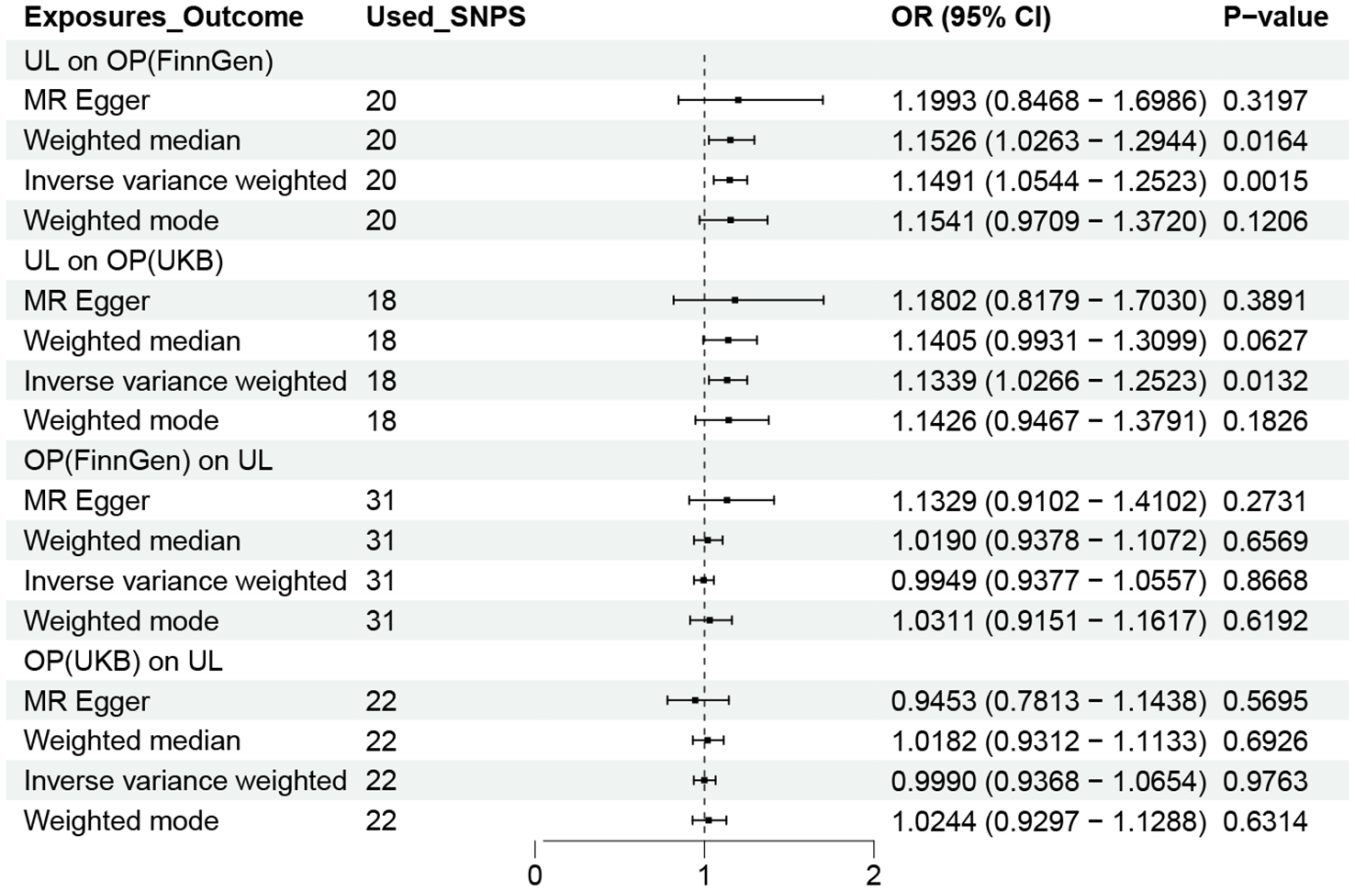
OR plot for UL and OP. OR, odds ratio; CI, confidence interval; SNPs, single-nucleotide polymorphisms; UL, urolithiasis; OP, osteoporosis.

Following a rigorous evaluation process, we selected suitable SNPs as instrumental variables (IVs) to fulfill three crucial assumptions. Twenty-three SNPs in all showed a strong correlation with UL. The F statistic for every variant were much > 10 in our study. Genetically predicted UL was related to OP (FinnGen: OR = 1.1491, 95% CI 1.0544-1.2523; UKB: OR = 1.1339, 95% CI 1.0266-1.2523) (**Figure 2**). Consistent findings were also observed in the remaining three MR approaches.

#### 3.1.2 Causal effects of OP on UL

To acquire a suitable number of SNPs in reverse MR, we change the p value of OP to 5×10^-6^. However, no method was found to show a causal relationship between OP and UL, either in the FinnGen or UKB database (**Figure 2**).

### 3.2 Causal effects of UL on BMD

**Figure 3:**
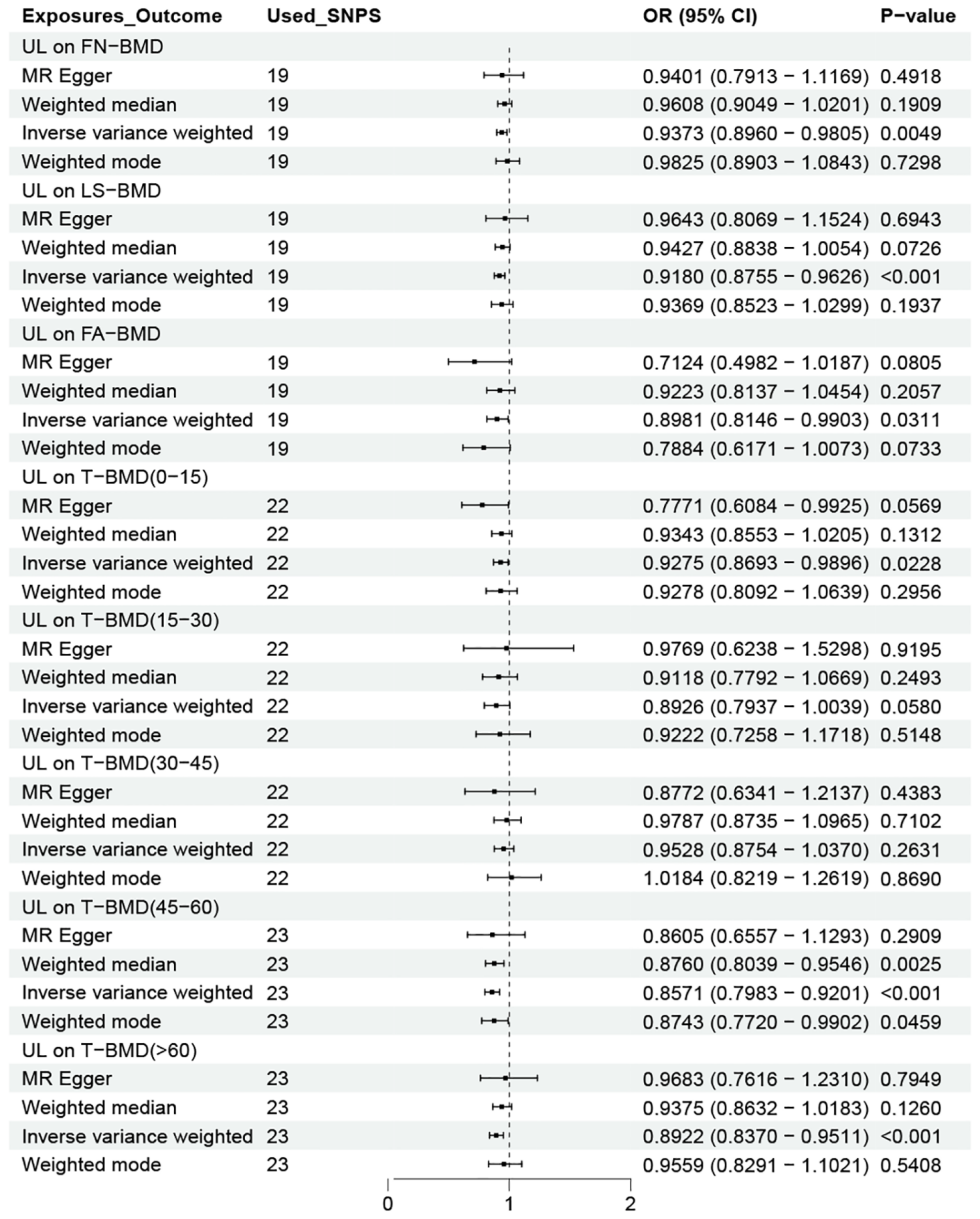
OR plot for UL and BMD levels at different ages and sites. OR, odds ratio; CI, confidence interval; SNPs, single-nucleotide polymorphisms; UL, urolithiasis; FN-BMD, femoral neck bone mineral density; LS-BMD, lumbar spine bone mineral density; FA-BMD, forearm bone mineral density; T-BMD, total bone mineral density.

After determining the effect of UL on OP, we continued to study the relationship between UL and bone mineral density levels in different age groups and different parts. As shown in **Figure 3**, gene-determined UL was significantly correlated with the decreasing trend of FN-BMD, LS-BMD, and FA-BMD (FN-BMD: OR = 0.9373, 95% CI 0.8960-0.9805; LS-BMD: OR = 0.9180, 95% CI 0.8755-0.9626; FA-BMD: OR = 0.8981, 95% CI 0.8146-0.9903). The remaining three methods yielded similar trends, even if some P values did not reach statistical significance. Similarly, the findings showed an increased risk of reduced BMD due to UL in addition to patients in the 15–45 year age group.

### 3.3 Two-step MR analyses

#### 3.3.1 Causal effects of UL on mediators

**Figure 4:**
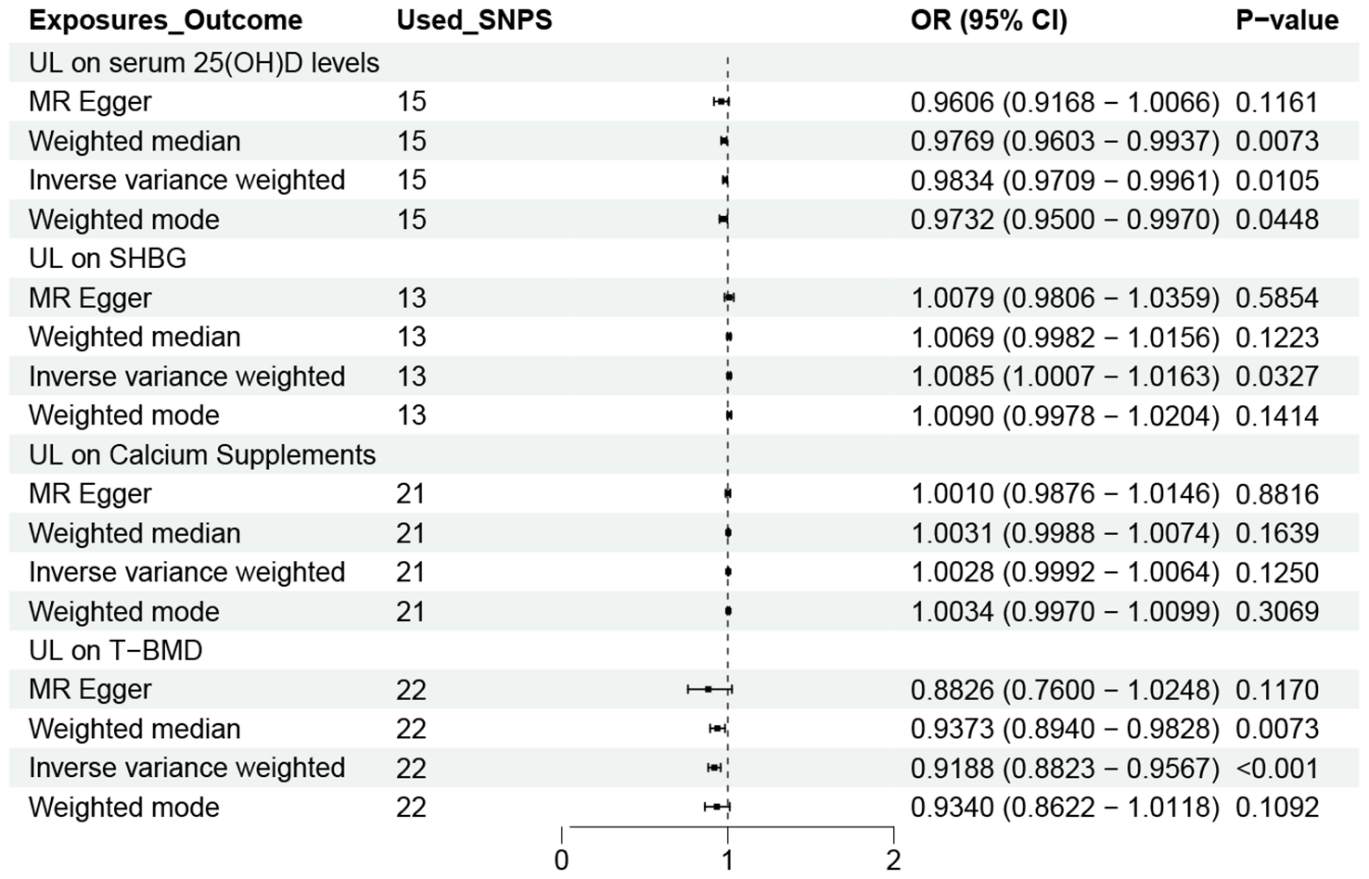
OR plot for UL and four Mediators. OR, odds ratio; CI, confidence interval; SNPs, single-nucleotide polymorphisms; UL, urolithiasis; T-BMD, total bone mineral density; SHBG, sex hormone binding globulin.

**Figure 4** shows the causal effect of UL on the four possible mediations obtained using the four MR methods. As we can see that UL was negatively correlated with serum 25 (OH) D (OR=0.9834, 95% CI 0.9709-0.9961) and T-BMD (OR=0.9188,95% CI 0.8823–0.9567) levels in the IVW method. Our results also revealed a positive correlation between UL and SHBG levels(OR=1.0085,95% CI: 1.0007–1.0163). However, we did not find a causal relationship between UL and calcium supplements. So, we did not analyze it in the next step.

#### 3.3.2 Causal effects of possible mediators on OP

**Figure 5:**
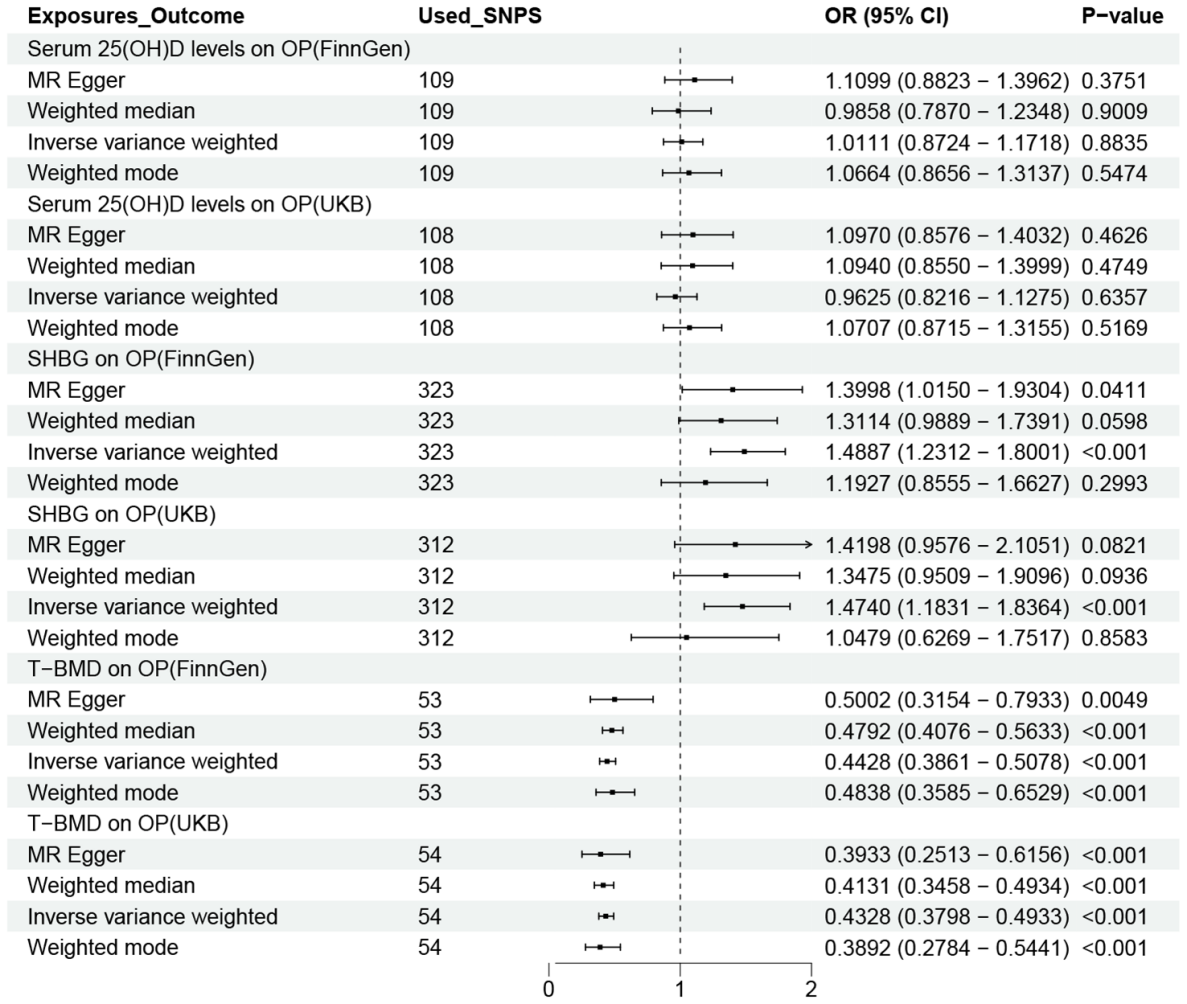
OR plot for the three possible Mediators and OP. OR, odds ratio; CI, confidence interval; SNPs, single-nucleotide polymorphisms; UL, urolithiasis; OP, osteoporosis; T-BMD, total bone mineral density; SHBG, sex hormone binding globulin.

**Figure 5** shows the causal effects of three possible mediators on OP in the FinnGen and UKB databases. As we can see that in the IVW method, SHBG (FinnGen: OR = 1.4887,95% CI 1.2312-1.8001; UKB: OR = 1.4740,95% CI 1.1831-1.8364) and T-BMD (FinnGen: OR = 0.4428,95% CI 0.3861-0.5078; UKB: OR = 0.4328,95% CI 0.3798-0.4933) were significantly correlated with OP. However, we did not observe a link between serum 25 (OH) D and OP. Based on the above analysis results, we calculated the mediating effect ratio of SHBG and T-BMD levels (**Table 1**).

**Table 1:** The mediation proportion of mediators in the causal relationship between UL and OP.

Mediators	Beta	Beta1	Beta2	Mediated Proportion (%)
T-BMD	0.1389 (FinnGen)	-0.0847	-0.8146	49.67%
	0.1256 (UKB)		-0.8374	56.47%
SHBG	0.1389 (FinnGen)	0.0084	0.3979	2.406%
	0.1256 (UKB)		0.3880	2.595%
Serum 25 (OH)D	0.1389 (FinnGen)	-0.0167	0.0110	–
	0.1256 (UKB)		-0.0383	
Calcium supplements	0.1389 (FinnGen)	0.0028	–	–
	0.1256 (UKB)			

Beta was the overall impact of UL on osteoporosis. Beta1 was the impact of UL on the mediators, and Beta2 was the effect of the mediators on OP.

### 3.4 Sensitivity analyses

We evaluated heterogeneity based on the results of the Cochran ‘s Q test. The importance of IVW estimates remains following normalization using a multiplicative random effects model, even though several of our results show high heterogeneity. Furthermore, we determine whether there is pleiotropic using the p value that corresponds to the MR-Egger intercept (**Supplementary Table S6**). In our analysis to correct horizontal pleiotropy, the MR-PRESSO global test is utilized to solve heterogeneity and detect and reject outliers. In all our positive results, the results that remain after these outliers are eliminated are consistent with the initial findings. The causal reasoning of the primary analysis is strengthened by the consistency of the sensitivity analysis.The sensitivity analysis outcomes are presented within **Table 2**. As depicted in **Table 2**, our sensitivity analysis reveals the presence of heterogeneity but not horizontal pleiotropy. For the results exhibiting heterogeneity in our study, we utilized the random-effects IVW model as the principal analytical approach.

**Table 2:** The results of sensitivity analysis.

Outcome	Exposure	Pleiotropy		Heterogeneity			
		Egger Intercept	P	QIVW	PIVW	QEgger	PEgger
OP (FinnGen)	UL	-0.0042	0.8064	10.9025	0.9271	10.8407	0.9010
OP (UKB)	UL	-0.0041	0.8270	13.8212	0.6797	13.7718	0.6157
UL	OP (FinnGen)	-0.0130	0.2369	25.6733	0.6917	24.2146	0.7183
UL	OP (UKB)	0.0070	0.5521	27.2236	0.1636	26.7345	0.1429
FN-BMD	UL	-0.0003	0.9723	21.9498	0.2342	21.9482	0.1867
FA-BMD	UL	0.0240	0.2051	25.6219	0.1087	23.2472	0.1414
LS-BMD	UL	-0.0051	0.5816	17.7856	0.4699	17.4615	0.4235
T-BMD (0–15)	UL	0.0177	0.1571	20.4757	0.4913	18.3148	0.5667
T-BMD (15–30)	UL	-0.0091	0.6868	21.9808	0.4006	21.7984	0.3515
T-BMD (30–45)	UL	0.0082	0.6105	25.5878	0.2226	25.2498	0.1920
T-BMD (45–60)	UL	-0.0004	0.9762	37.3749	0.0215	37.3733	0.0152
T-BMD (>60)	UL	-0.0081	0.4955	33.9245	0.0500	33.1645	0.0444
serum 25 (OH)D	UL	0.0024	0.3261	15.8026	0.3256	14.6305	0.3310
SHBG	UL	0.0001	0.9661	20.0496	0.0662	20.0461	0.0447
Calcium supplements	UL	0.0002	0.7949	28.3406	0.1016	28.2373	0.0790
T-BMD	UL	0.0040	0.5892	37.0573	0.0166	36.5075	0.0134
OP (FinnGen)	serum 25 (OH)D	-0.0033	0.3010	121.1275	0.1829	119.9168	0.1854
OP (FinnGen)	SHBG	0.0037	0.0681	349.6864	0.1069	346.0292	0.1259
OP (FinnGen)	T-BMD	-0.0066	0.5894	93.1756	0.0004	92.6398	0.0003
OP (UKB)	serum 25 (OH)D	-0.0047	0.1777	109.0927	0.4256	107.2305	0.4483
OP (UKB)	SHBG	0.0006	0.8221	392.9538	0.0011	392.8897	0.0010
OP (UKB)	T-BMD	0.0051	0.6633	71.8528	0.0433	71.5888	0.0371

## 4 Discussion

By combining a bidirectional two-sample and two-step MR analysis, we investigated the link between UL and OP. The findings of gene prediction show that UL increases the risk of developing OP. Causality is not bidirectional, as our results did not support a causal effect of OP on UL. In addition, we noticed that the impacts of UL on BMD varied depending on the age group. Specifically, UL was not associated with BMD levels in patients aged 15-45 years. This age-dependent phenomenon has important implications for clinical practice. This may be related to the different manifestations of bone mass growth at various ages, as mentioned above. Our study again highlights age as a critical component in OP (41, 42). Moreover, UL has a causal effect on FN-BMD, LS-BMD, and FA-BMD. Mediation analysis showed that in UL patients, bone mineral density (mediator ratio: FinnGen:49.68%; UKB:56.45%) and SHBG (mediator ratio: FinnGen:2.406%; UKB:2.595%) levels partially mediated the risk of osteoporosis, indicating that these two mediators may be involved in the mechanism by which UL induced OP. These findings broaden the understanding of the link between UL and OP.

Our findings regarding the causal relationship between UL and OP confirm previous epidemiological studies (9, 43–48). However, in the reverse MR analysis, there was no causal evidence to support the idea that OP increases UL risk, which is in some contradiction with several published studies (49–53). A case–control study of 135,622 adults aged over 40 years showed a positive association between UL and OP (8), which is consistent with the findings of our study. But age was not taken into account when stratifying patients in this investigation, leaving uncertainty about the causal relationship between UL and OP in all age groups. Therefore, we investigated the causal relationship of UL on BMD at different ages. In addition, the study conducted a two-way analysis showing that adults with OP had a higher risk of UL. Similarly, a cumulative analysis also demonstrated a 1.51-fold increased risk of kidney stones in patients with OP compared with healthy people without OP (52). However, in our MR analysis, our results did not support the causal effect of OP on UL, which may be due to the effects of various confounding factors on observational studies. Therefore, more epidemiological studies are necessary to accurately assess the relationship between OP and UL.

The biological mechanism underlying the relationship between UL and OP remains unknown. The interplay of genetic and environmental factors forms the basis of all disease occurrences. The majority of clinical studies have reported the mediating role of lifestyle and environmental factors, such as diet and medication (54–57). However, this explanation fails to clarify the complications of osteoporosis in patients with UL who have normal diet and urinary calcium levels (58, 59). Therefore, the emergence of OP in patients with UL is not solely orchestrated by external factors such as dietary choices and physical activity. To further explore the mechanisms by which urolithiasis leads to OP, we conducted a mediation analysis. Mediation analysis indicated that the genetic predictive effect of UL on OP is mediated by T-BMD and serum SHBG levels. Previous research has not reported on this. This finding both confirms and expands upon the results of the TS-MR analysis. BMD has been identified as a key factor in osteoporosis and do not be detailed here. This study also found that an increase in SHBG leads to an increased risk of osteoporosis. Idiopathic osteoporosis (IOP) is considered a subgroup of osteoporosis with no clear etiology. A recent meta-analysis identified that an increase in SHBG may contribute to idiopathic osteoporosis, further corroborating our findings (60). The study compared bone density, hormone levels, and bone turnover markers between patients with IOP and healthy controls, revealing that SHBG levels were elevated in patients with IOP compared to the healthy control group. However, due to limitations in the database, we were unable to obtain GWAS data for specific types of osteoporosis for further analysis. The precise biological pathways through which SHBG influences OP risk are yet to be fully elucidated, but several hypotheses have emerged. As a glycoprotein, SHBG has the capacity to bind with sex hormones, including testosterone and estradiol, thereby modulating their bioavailability (61). This effect may affect bone metabolism by altering the levels of free sex hormones. Specifically, Testosterone is known to promote bone formation, whereas estradiol curbs the resorption process (61). Thus, elevated SHBG levels might decrease the availability of sex hormones, leading to heightened bone turnover and a reduction in bone mineral density (61, 62). An additional mechanism could be the direct influence of SHBG on osteoblasts and osteoclasts, as it has been observed to bind to specific receptors on these cells, potentially impacting their functionality and activity (14). Additionally, we explored the mediating effects of serum 25(OH)D levels and calcium supplementation. We found a causal association between UL and serum 25 (OH) D levels, even though we did not detect a mediation function between serum 25 (OH) D levels and calcium supplements. 25(OH) D is the final metabolic product of Vitamin D (12). Serum 25(OH)D binds to vitamin D-binding protein (VDBP) and albumin in peripheral blood, with only a small fraction existing in an unbound, free form. However, according to the research by M. S. Johnsen et al, significant correlations with bone mineral density are observed only in measurements of free and bioavailable 25(OH)D (63). Since the calculation of free and bioavailable 25(OH)D depends on the binding coefficients of VDBP, adjusting for VDBP phenotypes could directly impact the bioavailability and functionality of vitamin D. But we did not discover a link between serum 25 (OH) D levels and OP. Therefore, whether UL causes OP by lowering serum 25 (OH) D levels deserves further investigation.

There are several advantages in our MR study. To begin with, this is the first study that, to the best of our knowledge, assesses the relationship between UL and OP using a bidirectional two-Sample and two-step mendelian randomization study. When MR analysis was used, potential bias such as reverse causation and confounders could be successfully reduced, strengthening the causal inference. Second, to avoid population overlap by selecting exposure and outcome data from different databases. In addition, the GWAS data of OP from the UKB database were selected to validate our results. Third, a variety of methods were used for MR analysis and exclusion of heterogeneity and pleiotropic analysis. Meanwhile, our study also has some flaws and shortcomings. Initially, we were unable to locate datasets with serum free 25 (OH) D levels in public databases and were unable to run mediation analysis. In addition, despite rigorous attempts to address pleiotropy, the complete elimination of all forms of pleiotropic effects in MR studies remains unattainable. Therefore, there may be unexplored pathways and confounding variables that could bias the results. Furthermore, a significant limitation of this study is the inability to stratify the analysis by disease severity, as well as other key factors such as age, which may provide a more nuanced understanding of the associations studied. Finally, sex-specific aggregated data on GWAS associated with disease are not available.

## 5 Conclusion

In conclusion, the study establishes a direct causal link between urolithiasis and OP, independent of environmental factors. Furthermore, mediation analysis showed that bone density and SHBG levels partially mediated the risk of OP in UL patients, suggesting that both mediators may be involved in the mechanism of UL-induced OP. These findings broaden the understanding of the link between the UL and the OP. Thus, regardless of lifestyle, urolithiasis patients should remain vigilant about the risk of OP and consider regular OP screening.

## Data Availability

The original contributions presented in the study are included in the article/**Supplementary Material**. Further inquiries can be directed to the corresponding author.
